# Alström syndrome—wide clinical variability within the same variant: a case report and literature review

**DOI:** 10.3389/fped.2024.1463903

**Published:** 2024-09-25

**Authors:** Diana Jecan-Toader, Adrian Trifa, Bogdan Lucian, Tudor Lucian Pop, Simona Sorana Cainap

**Affiliations:** ^1^ Medical Oncology Discipline, Department of Oncology, Iuliu Hatieganu University of Medicine and Pharmacy, Cluj-Napoca, Romania; ^2^ 2nd Pediatric Clinic, Emergency Clinical Hospital for Children, Cluj-Napoca, Romania; ^3^ Discipline of Medical Genetics; Center for Research and Innovation in Personalized Medicine of Respiratory Diseases, “Victor Babes” University of Medicine and Pharmacy, Timisoara, Romania; ^4^ Center of Expertise for Rare Pulmonary Diseases, Clinical Hospital of Infectious Diseases and Pneumophysiology “Dr. Victor Babes” Timisoara, Romania; ^5^ Breast Cancer Center, The Oncology Institute “Prof. Dr. Ion Chiricuta”, Cluj-Napoca, Romania; ^6^ Pediatric Department, “Dr. Constantin Opris” Emergency County Hospital, Baia Mare, Romania; ^7^ 2nd Pediatric Discipline, Department of Mother and Child, Iuliu Hatieganu University of Medicine and Pharmacy, Cluj-Napoca, Romania

**Keywords:** Alström syndrome, dilated cardiomyopathy, cone-rod dystrophy, obesity, genotype-phenotype correlations

## Abstract

**Background:**

Alström disease is a rare disorder caused by various variants in the ALMS1 gene. It is characterised by multiorgan involvement, namely neurosensory deficits, endocrine and metabolic disturbances, cardiomyopathy, and hepatic and renal dysfunction. The disease exhibits marked interindividual variability, both in clinical manifestations and age of onset. Several attempts have been made to establish a relationship between phenotype and genotype, with little success.

**Methods:**

We present the case of an infant who presented with dilated cardiomyopathy, above-average weight and neurosensory deficits, raising the suspicion for Alström syndrome, later confirmed through genetic testing. Moreover, we conducted an extensive literature search to identify all reported cases having the same variant as our patient, in order to evaluate whether specific mutated alleles have a role in determining phenotype-genotype associations.

**Results:**

A 4-month-old female infant with a recent history of bronchiolitis was referred to our centre due to a systolic murmur. In our service, the clinical exam was significant for above-average weight, dyspnea, wheezing and a grade II systolic murmur. Echocardiography revealed dilated cardiomyopathy with severe systolic dysfunction of the left ventricle. Laboratory investigations revealed elevated NT-proBNP and troponin levels, along with positive IgM antibodies for CMV and EBV. Dilated cardiomyopathy attributed to viral myocarditis was suspected. Treatment with ACE inhibitors and diuretics was started, with a favourable response initially. However, after a few months, the patient presented with vertical nystagmus and head bobbing. The ophthalmologic exam revealed cone-rode dystrophy. Considering the constellation of symptoms, Alström syndrome was suspected. Genetic testing revealed a homozygous variant [c.4156dup (p.Thr1386Asnfs*15)] in the ALMS1 gene, confirming the diagnosis.

**Conclusion:**

Our literature review revealed 8 additional cases harbouring the same variant as our patient, five in a heterozygous state, two in a homozygous state and one with only one allele identified. The identified patients presented high heterogeneity of clinical manifestations and age of onset. The heterogeneity persisted even in patients with homozygous variants, suggesting the involvement of factors beyond the specific disease-causing variant in determining disease manifestation. Therefore, genotype-phenotype correlations might not be supported by specific variants.

## 1 Introduction

Alström syndrome is a rare recessive genetic disorder caused by pathogenic variants in the ALMS1 gene, located on the short arm of chromosome 2 (1). To date, 388 variants in the ALMS1 gene have been identified (2). The ALMS1 molecule is a large protein composed of 4,169 amino acids and its functions are still not fully understood (3). Several studies have linked the ALMS1 protein to primary cilia and centrosome function, as well as endosome recycling (1, 3–5). Moreover, a recent study has identified important inhibition of TGF-beta signalling in the pathophysiology of the disease, accounting for the generalised fibrosis frequently observed in patients (5). The widespread expression of the protein across various tissues explains the extensive organ involvement observed in the disease (6).

Alström syndrome is characterised by multi-organ involvement, among which neurosensory deficits such as cone-rode dystrophy and hearing loss are cardinal features. Additionally, the syndrome presents metabolic and endocrine disturbances, namely type II diabetes, hyperlipidaemia, hypothyroidism, obesity, and short stature, as well as complications such as cardiomyopathy, recurrent pulmonary infections, fatty liver disease, cirrhosis and renal dysfunction (6, 7). The disease manifests across a heterogeneous spectrum, with both age of onset and severity of clinical symptoms varying between patients (6, 8). Several studies have attempted to identify a genotype-phenotype correlation with little success (2, 9–11). However, all studies have investigated the associations between phenotype and the location of the variant in the gene. It is plausible to assume that certain individual mutant alleles may have an impact on the phenotype of the disease.

We present the case of an infant with dilated cardiomyopathy and above-average weight gain that later developed vertical nystagmus, raising the suspicion for Alström syndrome. Genetic testing revealed a homozygous disease-causing variant in the ALMS1 gene. Furthermore, we conducted a thorough literature review to identify all the reported cases sharing the same variant as our patient. This review aims to investigate potential genotype-phenotype associations arising from the shared mutant allele.

## 2 Case report

A 4-month-old baby girl was referred to our centre for the investigation of a systolic murmur. One month before being brought to our centre, the patient presented important respiratory distress, prompting the parents to seek medical attention. The clinical exam detected a systolic murmur, wheezing and crackles, along with a decrease in oxygen saturation up to 93% in room air. The chest x-ray revealed pulmonary hyperinflations and bilateral disseminated opacities. Echocardiography revealed left ventricular hypertrophy, without any other changes. The case was interpreted as bronchopneumonia and antibiotic and symptomatic treatment was started, under which the patient's evolution was favourable. However, dyspnoea persisted, prompting the parents to seek further investigations at our centre.

The anamnesis did not reveal any familial history of heart disease. The baby girl was born to nonconsanguineous parents following a pathological pregnancy characterised by maternal arterial hypertension. The birth occurred at term, with a birth weight of 3,000 g and normal adaptation to extrauterine life.

In our service, the physical exam was notable for above-average weight, wheezing, mild respiratory distress, grade II systolic murmur and mild hepato-splenomegaly. The laboratory investigations revealed mild lactic acidosis, and normal ranges for blood parameters, inflammatory markers, hepatic and renal function. Notably, cardiac markers were elevated, with high troponin levels (240.9 pg/ml) and markedly increased NT-pro-BNP levels (2,449 pg/ml). Thoracic radiography was unremarkable. Echocardiography revealed dilated left cavities of the heart, severe mitral regurgitation, and significant systolic dysfunction. Subsequent cardiac magnetic resonance (MRI) confirmed dilated cardiomyopathy, with an estimated ejection fraction of the left ventricle of 36%. Additionally, it identified a linear late gadolinium enhancement area, from the base of the heart to its apex, indicative of fibrosis. We investigated aetiologies for dilated cardiomyopathy, excluding arrhythmogenic and metabolic causes, intracardiac shunts and anomalous origin of coronary arteries. However, viral serology revealed positive IgM antibodies for Cytomegalovirus (CMV) and Epstein-Barr virus (EBV).

The case was interpreted as dilated cardiomyopathy with moderate systolic dysfunction, likely attributed to viral myocarditis. Treatment was started with diuretics (Furosemide at 2 mg/kg/day and Spironolactone at 2 mg/kg/day), Angiotensine-converting enzyme (ACE) inhibitors (Captopril at 0.5 mg/kg/day) and Carnitine supplementation (10 mg/kg/day), with rapid alleviation of dyspnoea and wheezing. During hospitalisation, a decline in IgM antibody titters for CMV and EBV was noted, accompanied by the seroconversion to IgG antibodies for CMV and an elevation in the IgG antibody titters for EBV, further supporting the suspicion of a viral aetiology. Over the next few months, the left ventricle ejection fraction gradually improved, and cardiac markers returned to normal values. Notably, the patient consistently maintained an above-average weight.

**Figure 1:**
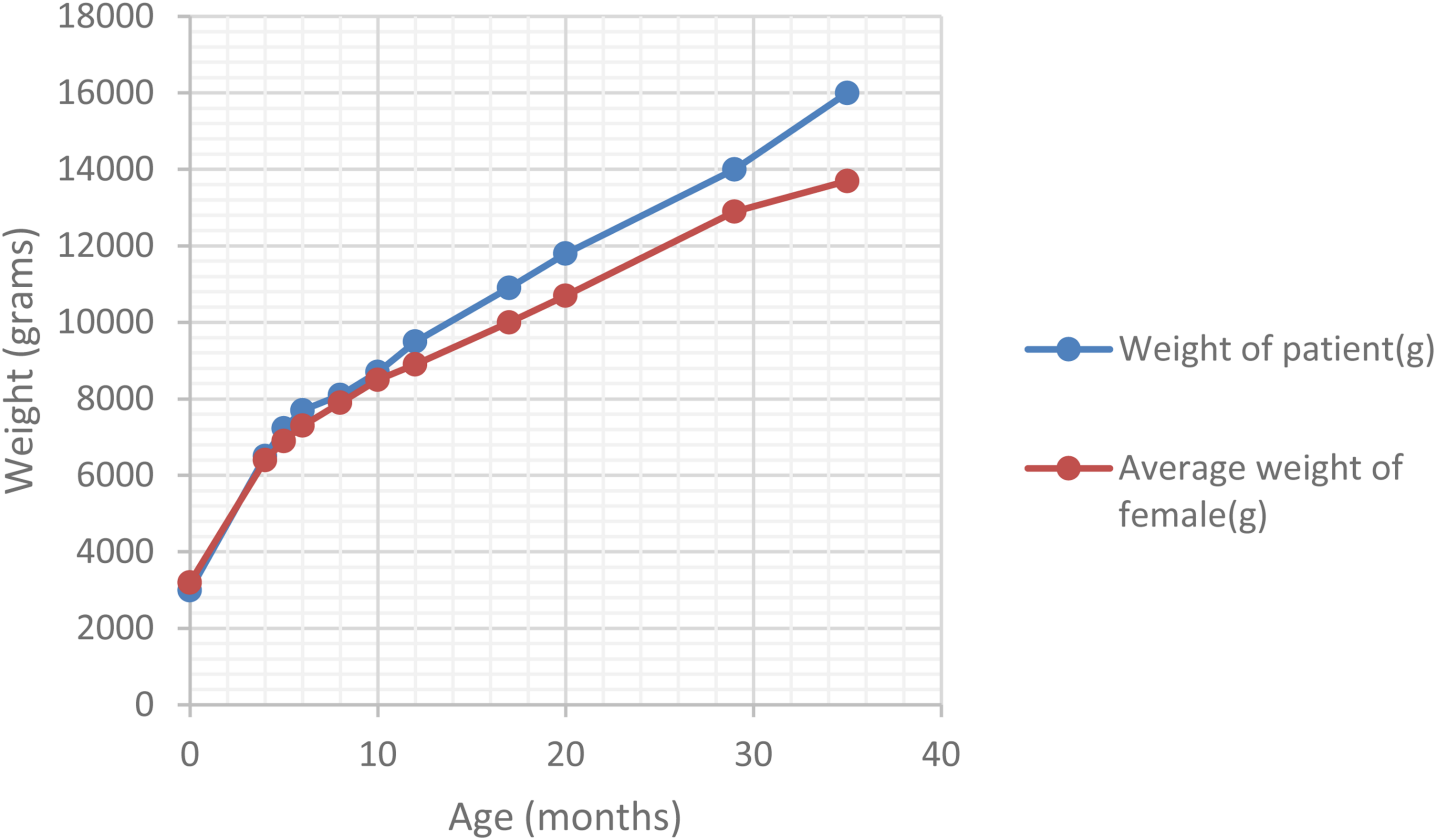
Comparison of ponderal curve in our patient and the average female infant.

At the 6-month follow-up, the patient presented vertical nystagmus, head bobbing and right torticollis. Neurological causes for the symptoms were excluded. The trans-fontanellar ultrasound and resting-state EEG did not reveal any pathological changes. The MRI of the head yielded unremarkable findings. Ophthalmologic assessment revealed cone-rod dystrophy. The triad of persistent above-average weight (Figure 1), dilated cardiomyopathy, and cone-rod cell dysfunction raised the suspicion of Alström syndrome. Genetic testing was carried out in a third-party laboratory (Invitae, San Francisco, CA, USA - Ciliopathies Panel: 174 genes), using sequence analysis and deletion or duplication testing of genes. It confirmed the diagnosis, identifying a homozygous disease-causing variant [c.4156dup (p.Thr1386Asnfs*15)] in exon 8 of the ALMS1 gene. The variant had been classified as pathogenic using the Sherloc classification criteria (ClinVar accession - RCV000192391.14).

**Figure 2:**
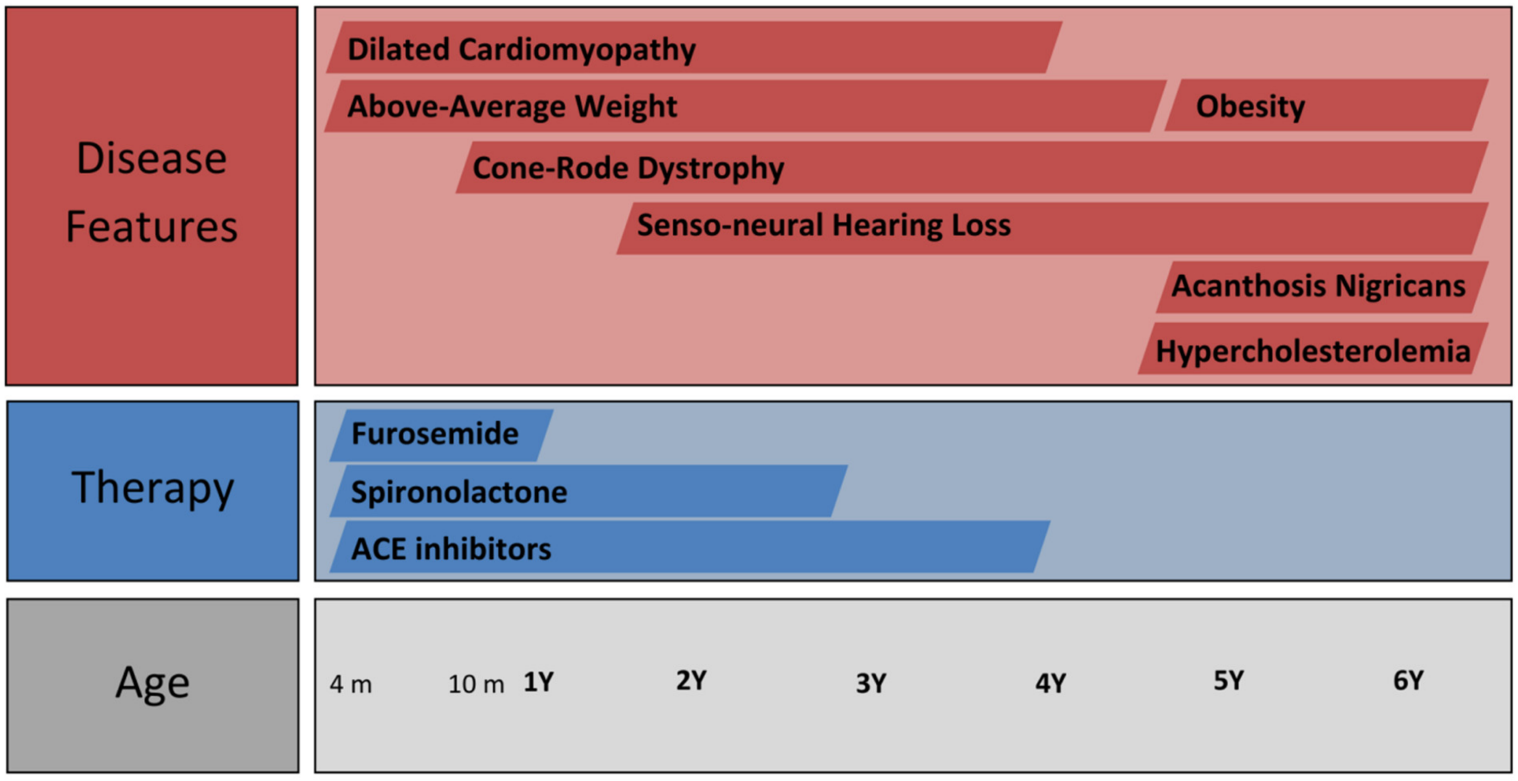
Timeline of the case including disease features and therapy.

**Figure 3:**
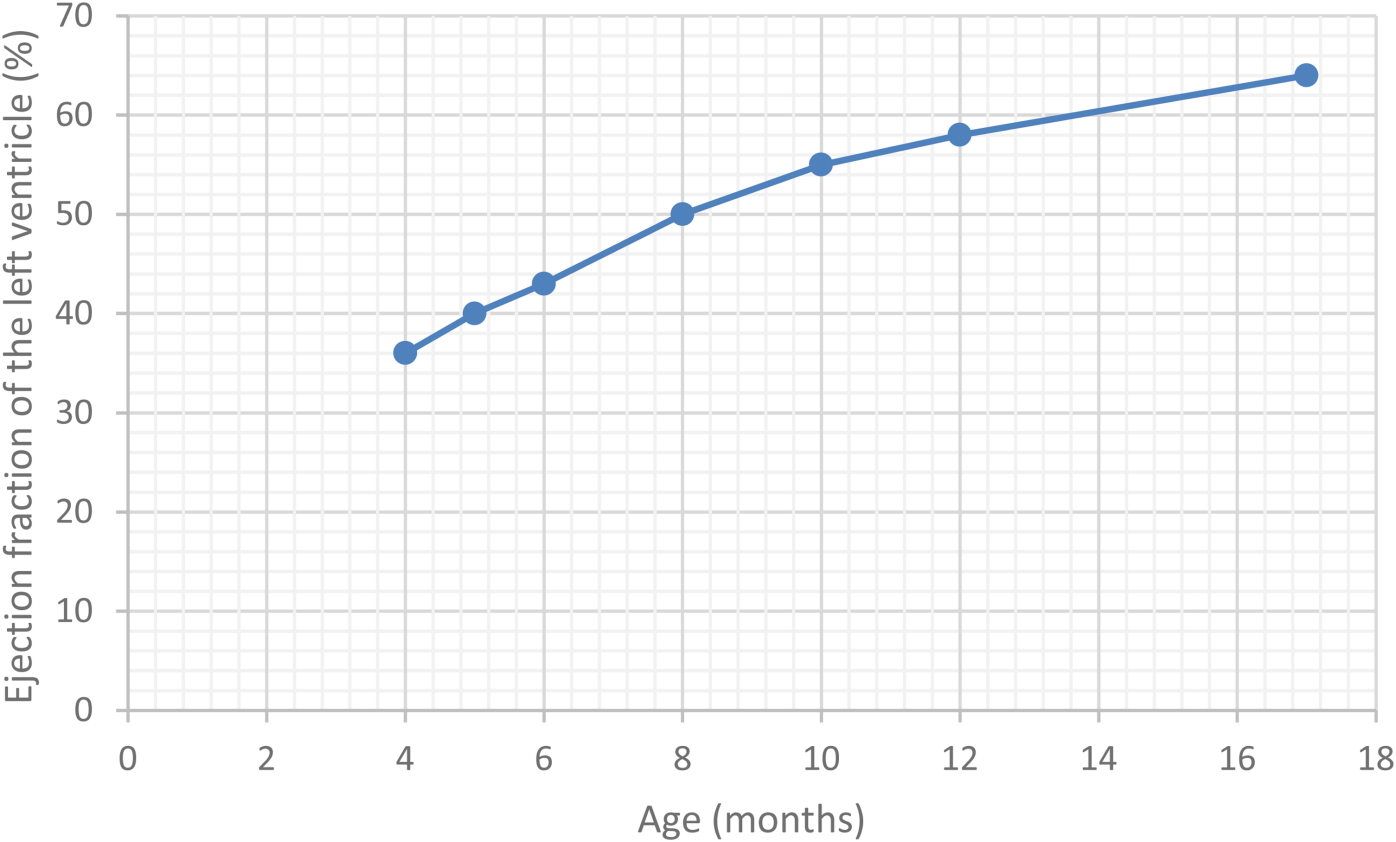
Timeline of the ejection fration of the left ventricle in our patient.

Over the subsequent years of follow-up, the patient developed senso-neural hearing loss and the vision loss was exacerbated, as well as presenting accelerated weight gain. The case timeline is presented in Figure 2. However, the cardiac function normalised (Figure 3), leading to the complete cessation of cardiac treatment by the age of 4. At the age of 5, the clinical examination revealed above-average weight (33 kg, *Z* score = 2.70) and normal height for age, but a BMI of 26.3 (percentile 100%, *Z* score = 2.86), indicative of extreme obesity. Additionally, the examination identified acanthosis nigricans. This prompted laboratory investigations for metabolic syndrome, which revealed glycated haemoglobin consistent with prediabetic values (6.3%) with normal fasting glycaemia, as well as hypercholesterolemia, slightly raised GGT levels and normal hepatic, renal and thyroid function. A multi-disciplinary follow-up plan was developed in cooperation with the patient's parents to ensure correct follow-up of the disease progression over the next years.

## 3 Discussion

Alström syndrome is a very rare monogenic ciliopathy, its incidence being estimated at around 1 in 50,000–100,000 people (7). It is caused by pathogenic variants in the ALMS1 gene, with 388 disease-causing variants reported to date (2). Because of the ubiquitous expression of the ALMS1 protein in tissues, the disease affects numerous organs and systems (6). Sensory complications are frequent, with almost all patients developing retinal dystrophy within the first year of life. The first symptoms are photophobia and horizontal nystagmus, with most patients progressing to complete cecity by the second decade of life (7, 8). Hearing loss usually develops within the first decade of life, with about 10% progressing to complete deafness (7, 8, 12). Cardiac manifestations are common, with a reported incidence of dilated cardiomyopathy varying between 30%–60% at different life periods (10, 13). Most of the patients present with heart failure caused by severe dilative cardiomyopathy in the first months of life. The pathophysiology of the dilated cardiomyopathy is still unknown. The survivors usually recover cardiac function within the first years of life (usually by the age of 3), maintaining a normal-low cardiac function for several years (8, 10). Additionally, there have been reports of patients with Alström syndrome developing mitogenic cardiomyopathy, an extremely rare form of cardiomyopathy leading to death in early infancy (14, 15). Since ALMS1 protein has a role in centrosome organisation during mitosis, its deficiency compromises post-natal cardiomyocyte cycle cell arrest, contributing to the development of mitogenic cardiomyopathy (15). It has been hypothesised that mitogenic cardiomyopathy might be extreme form of the infantile dilated cardiomyopathy seen in the disease (16). In adolescence and young adulthood, congestive heart failure can reoccur in the form of restrictive cardiomyopathy caused by diffuse interstitial fibrosis (8, 10, 17). Obesity is a nearly ubiquitous feature, becoming apparent around 6 months to 1 year of age and sometimes improving after puberty (8). Insulin resistance and hyperinsulinemia represent early metabolic alterations, in some cases developing before the occurrence of obesity. Most patients develop type II diabetes, with great variability in the age of onset (8). Other endocrine complications include hypertriglyceridemia, metabolic syndrome, short stature, hypothyroidism, female hyperandrogenism and hypogonadism in males (18). Renal manifestations present great variability in onset age and progression rate (8, 19). End-stage renal disease can be caused by interstitial fibrosis, glomerular hyalinosis, and tubular atrophy, probably accentuated by arterial hypertension and diabetes (19). Steatotic liver disease and cirrhosis have been reported as hepatic complications of the disease (7, 8, 20). Recurrent otitis media, sinus and pulmonary infections have also been reported (21). Even though the majority of patients have normal intelligence and development, neurologic and psychiatric complications such as epilepsy, cerebellar anomalies, autistic or psychotic behaviour, obsessive-compulsive disorder or major depression have been reported (6).

Clinical manifestations are characterised by marked variability between individuals affected by the disease. Several studies have tried to establish an association between the phenotype and the location of the pathogenic variants in the ALMS1 gene. A small study on 16 patients by Bond et al. did not identify any associations between the location of the disease-causing variant and clinical manifestations (22). Another study on a cohort of similar size did not find any genotype-phenotype correlations (23). Marshall et al. have found a significant correlation between disease-causing variants in exon 8 of the ALMS1 gene and absent, delayed or milder renal manifestations (9). The study also identified a suggestive association between variants in exon 16 and the occurrence of infantile retinal degeneration, type II diabetes, cardiomyopathy and urological dysfunction (9). Another study on 19 patients has found that all individuals with milder phenotypes have disease-causing variants localised before exon 8 (11). A meta-analysis on genotype-phenotype correlation on a total of 357 patients has uncovered a relationship between variants in exon 10 and higher rates of hepatic dysfunction. However, the study concluded that the location of the disease-causing variant has little effect on the disease manifestations (2). Advocating for this result, a study conducted by Chen et al. suggests that milder phenotypes might not be associated with the variant site, but rather with the residual ALMS1 protein expression in cells (24).

Considering the weak association between the location of the variant in the ALMS1 gene and phenotype, it has been suggested that allelic effects might have an impact on disease manifestations (1, 9). However, the allelic effects are difficult to assess considering the rarity of the disease, as well as the high number of disease-causing variants described. To address this issue, we conducted a thorough review of the literature in order to identify all the reported cases with the same disease-causing variant as our patient. Our objective was to establish whether a discernible phenotype-genotype association exists for the c.4156dup (p.Thr1386Asnfs*15) sequence. This variant is predicted to cause premature translational stop signal, through a frameshift phenomenon, resulting in abnormal protein function determined by nonsense-mediated decay. It is known that loss-of-function variants are pathogenic in Alström syndrome (25). We conducted a comprehensive literature search across PubMed, Web of Science and ClinVar from inception to February 2024. The search strategy included the following terms: Alstrom syndrome, ALMS1 gene, exon 8, c.4156dup and p.Thr1386Asnfs*15. We examined the references of the included and other pertinent studies in order to identify other relevant studies. Studies were included if they reported on patients with the c.4156dup (p.Thr1386Asnfs*15) variant and provided clinical features. Our search identified nine patients, including our own, harbouring this variant: five in a compound heterozygous state, three in homozygosity and one with only a single allele identified. Data about ethnicity, age, gender and disease manifestations were extracted from the identified case reports. The results are presented in Table 1.

**Table 1:** Clinical and paraclinical characteristics of Alström patients with c.4156dup (p.Thr1386Asnfs*15) mutation.

Study reference	Country	Gene variant	Exon	Age	Sex	Visual dysfunction (since)	Senso-neurinal hearing loss (since)	Overweight/obesity (since)	Type 2 diabetes (since)	Lipids	Cardiac (since)	Respiratory (since)	Neurological (since)	Hepatic (since)	Renal	Other endocrine issues(since)
Astuti et al. (26)	NR	compound heterozygote: c.4156dupA (p.Thr1386Asnfs*15) and c.11207C>A (p.Ser3736*)	8/16	NR	F	Yes	NR	Obesity	Impaired glucose tolerance (13 y)	NR	NR	NR	Micro crania, psychomotor delay.	NR	NR	NR
Kuburović et al. (27)	Serbia	compound heterozygote: c. 3163dupG (p.E1055Gfs × 4) and c.4156dupA (p.T1386Nfs × 15)	8/8	15 y	M	Yes (early childhood)	Yes (early childhood)	Yes (childhood)	yes	Hypertriglyceridemia	Dilated cardiomyopathy, decreased LVEF = 29% (NR - infancy complicated by failure to thrive), hypertension (15 y)	NR	Epilepsy, mental disability - IQ = 40	Raised liver enzymes, cholelithiasis	Yes	Hypogonadism, gynecomastia
Long et al. (28)	USA	compound heterozygote: c.4156insA, p.Thr1386fs*15 AND c.6436C>T, p.Arg2146*	8/8	15m	M	Nystagmus	NR	No	NR	NR	Dilated cardiomyopathy decreased LVEF = 25% (1 m), improved at 15m	NR	Normal	NR	NR	NR
Ozantürk et al. (13)	Turkey	compound heterozygote: c.4156insA (p.Thr1386Asnfs*15) AND c.10822C>T (p.Arg3608*)	8/8	28 y	M	Yes (2 y)	Yes (7 y)	No	Yes (12 y)	Hyperlipidaemia	Normal	Severe pulmonary disfunction	Global delay, autism	Yes, severe	Normal	Hypogonadism
Zmyslowska et al. (29)	Polish	Compound heterozygote:c.4157insA (p.Thr1386Asnfs*13) and c.7373_7377delCAGAT (p.Thr2458Lysfs*9)	8/8	2 y	M	Yes	No	Obesity	No	NR	Dilated cardiomyopathy (infancy)	NR	NR	NR	Normal	NR
Waldman et al. and Brofferioa et al. (30, 31)	White (USA)	c.4156dupA (p.Thr1386Asnfs) and not identified	8/	5 y	F	Yes	No	Obesity	Insulin resistance	Hypertriglyceridemia	Dilated cardiomyopathy (infancy)	NR	NR	NR	Normal	NR
Waldman et al. and Brofferioa et al. (30, 31)	White (USA)	Homozygous: c.4156insA (p.Thr1386Asnfs*15)	8/8	9 y	M	Yes	Yes	Obesity	Insulin resistance	Normal	Normal	NR	NR	NR	Yes	NR
Ozantürk et al. (13)	Turkey	Homozygous: c.4156insA (p.Thr1386Asnfs*15)	8/8	12 y	F	Vision loss (6 y)	No	Yes (NR)	Yes (10 y)	Normal	Dilated cardiomyopathy (NR)	NR	Mental delay	Raised liver enzymes	Urolithiasis	Early puberty
Current study	Romania	homozygous: c.4156dup (p.Thr1386Asnfs*15)	8/8	5 y	F	Yes (infancy)	Yes (early childhood), otitis media	Yes (infancy)	Impaired glucose tolerance (5 y)	Hypercholesterolemia	Dilated cardiomyopathy (infancy)	Recurrent episodes of respiratory infections	Normal	Normal	Normal	Normal

y, years; m, months; NR, not reported.

All patients had both variants on exon 8, except one that had one pathogenic allele on exon 16 and one for which the second disease-causing variant was not identified. Except for a patient in their second decade of life and one with unreported age, all patients were under 18, of which two were adolescents. All patients presented some degree of vision loss, consistent with literature findings (7, 8). Out of the 9 patients, four presented neuro-sensorial hearing loss, three had no manifestations and no information was provided for two. Obesity was present in 7 out of the 9 patients. However, one of the individuals who did not exhibit obesity was a 15-month-old baby, being plausible to assume that this complication might not have manifested yet. Furthermore, the second patient's normal weight at 28 years of age could be explained by reports that obesity can moderate after puberty (8). Nearly all included individuals had some degree of glucose metabolism alteration, while four of them presented altered lipid metabolism. Six patients presented cardiac complications, two presented no history of cardiac manifestations and information regarding one patient was not provided. Out of the patients with available information, 6 out 8 (75%) presented dilated cardiomyopathy, slightly higher than the proportion reported from other cohorts (30%–60%) (10, 13). It is noteworthy that four patients were documented with infantile dilated cardiomyopathy, while information was not provided for two individuals. However, not all patients with homozygous variants presented dilated cardiomyopathy (two out of three). Regarding other complications, three individuals presented with hepatic manifestations, three patients with renal complications and three patients with other endocrine abnormalities (early puberty, hypogonadism and gynecomastia). Even though intellectual disabilities, psychiatric and neurologic complications are reported as rare manifestations of Alström syndrome, four of the identified 9 patients presented such complications: one individual presented micro-crania and psychomotor delay, one exhibited epilepsy and mental disability, one presented global delay and autism, while the other also presented mental disability.

The identified patients presented high heterogeneity of manifestations. The presence of compound heterogenous variants in some individuals could explain the diversity of described symptoms, as the presence of different disease-causing variants on each allele could contribute to different phenotypes. However, the persistence of phenotype heterogeneity in the identified patients of similar age with homozygous variants raises intriguing questions about the relationship between phenotype and genotype.

Further exploring this observation, there have been reports of different disease phenotypes arising in the same family. For example, Hoffman et al. reported on four siblings with identical haplotypes (in three of the children; the fourth passed away before the genetic analysis could be conducted) that presented various courses of infantile cardiomyopathy. The most affected child presented with neonatal cardiomyopathy, necessitating a cardiac transplant, but unfortunately succumbed to the condition. The eldest exhibited infantile dilated cardiomyopathy, with improvement of the cardiac function by age one, but later presenting deterioration at 11 years old. The remaining siblings, aged 7 and 8, also presented with infantile cardiomyopathy, which was stable in one child and resolved in the other (32). Variable clinical course was also reported by Hamamy et al. in four older siblings with the same haplotype (33). Another case report presents two siblings bearing the same homozygous variant with different clinical courses of dilated cardiomyopathy. The older sibling of four years of age showed improvement in his cardiac function, while the youngest, aged three, presented deteriorating cardiac performance despite maximal pharmaceutic support (34). Even identical twins can exhibit variable courses of the disease. In a report, monozygotic twins presented concordant courses for developmental delay and vision loss. However, one twin exhibited obesity and insulin resistance, while the other did not. Moreover, the twins manifested discordant courses of infantile dilated cardiomyopathy: while one presented rapid improvement, the other showed progressive cardiac dysfunction (35). Despite the observed clinical heterogeneity in individuals with the same variants, there is evidence that certain disease-causing variants might predispose to a more severe course of the disease. For example, a study has identified a specific disease-causing variant (c.7911dupC) as posing a high risk for a severe course of infantile dilated cardiomyopathy (10).

All the aforementioned observations suggest that apart from possible small variations attributed to the variant itself, the genotype-phenotype relationship in Alström syndrome might be largely affected by multiple factors, such as genetic and epigenetic modifiers, as well as environmental and infectious exposures. Novel research has identified potential gene modifiers responsible for variable expression or penetrance of monogenic disorders. Potential regulators might be cis- and trans-acting genetic modifiers. Variants in non-coding regions, such as promoters or enhancers, located on the same chromosome as the gene they affect, are called cis-acting genetic modifiers. They influence directly the expression of the target gene, potentially leading to dysregulations that affect multiple genes in the regulatory network. Trans-acting genetic modifiers are factors, usually proteins or RNA molecules codified in a different region of the genome that interact with cis-acting sequences in order to modify gene expression (36, 37). Castel et al. demonstrated that variants in cis-regulatory non-coding regions could enhance the expression of the pathogenic variant, potentially aggravating the phenotype (36, 38). Furthermore, recent studies have suggested that common background genetic variants might influence the phenotypic presentation of certain disorders, indicating that these genetic differences could have a cumulative effect on the expression of some pathogenic variants and disease severity (39, 40). Another potential mechanism for variable phenotype-genotype expression is genetic compensation through functional redundancy, where two different genes are responsible for the same function and one gene can effectively compensate for the inactivation of the other, minimizing the effect on the phenotype (41). Somatic mosaicism, caused by postzygotic *de novo* mutation, could also contribute to variability in the phenotype-genotype presentation. While individuals with mosaicism usually experience less severe forms of monogenic diseases, their phenotype is predominantly influenced by the tissues that contain the mutated variant, resulting in low or no penetrance (36). Epigenetic factors, such as DNA methylation, histone changes and non-coding RNAs might influence the phenotypic variation of Mendelian inherited diseases (42, 43). Moreover, environmental factors, such as diet, physical activity, *in utero* exposures, and toxin consumption could exert their influence on the epigenetic markup, thereby influencing the phenotype of the disease (36).

The interplay among these factors might lead to the diverse phenotypic spectrum observed in the disease, characterised by variable age of onset, clinical manifestations, and severity of the disease. Understanding the mechanisms behind the observed phenotypic heterogeneity is crucial for prognostic predictions and advancing precision medicine. Further research, studying the interactions between specific disease-causing variants, genetic and epigenetic modifiers, as well as environmental factors are necessary for advancing our understanding of the disease. Furthermore, it is important to note that our result might be influenced by potential biases caused by different reporting methods of the authors of included articles. This variability in reporting practices could impact the comprehensiveness of the available data, and, consequently, our conclusion. Therefore, big prospective studies, such as those facilitated by international registries, might provide robust evidence for phenotype-genotype associations within the same variant.

## 4 Patient perspective

The effects of visual deficits, hearing loss, cardiomyopathy and obesity have a great burden on the patient and their family. Understanding the genotype-phenotype associations in this disease holds great importance for clinicians, patients, and their families. It offers valuable insights into disease progression, helping the involved parties to cope with its challenges. This information aids the patient and the family prepare for what lies ahead, alleviating the uncertainty. It allows them and the clinician to stay one step ahead of the disease, enabling them to better prepare for possible clinical courses.

## Data Availability

The original contributions presented in the study are included in the article/Supplementary Material, further inquiries can be directed to the corresponding author.
